# Comparative Analysis of Pavement Performance–Environmental–Cost Nexus for Desulfurized Rubber Powder Composite SBS-Modified Asphalt Mixture

**DOI:** 10.3390/ma19132750

**Published:** 2026-06-27

**Authors:** Mingcheng Jing, Hui Dou, Chunyu Zhang, Liangying Li, Jing Li, Bo Li

**Affiliations:** 1 Gansu Road and Bridge Construction Co., Ltd., Lanzhou 730030, China; 2 Gansu Industry Technology Center of Transportation Construction Materials Research and Application, Lanzhou Jiaotong University, Lanzhou 730070, China

**Keywords:** comparative analysis, desulfurization rubber powder, modified asphalt, SBS, economic costs, carbon emissions

## Abstract

This study aims to systematically evaluate the balancing mechanism between road performance, carbon emissions, and economic cost when selecting asphalt materials for severe cold regions, filling the gap in multi-criteria decision-making for composite chemical modifications. To address alternating temperatures, heavy traffic, and modified asphalt transport difficulties, this study presents a novel evaluation framework focusing on the performance–environmental–cost nexus of a desulfurized rubber powder composite SBS-modified asphalt mixture, which provides a clear technological breakthrough for high-ratio scrap tire recycling in seasonal frost zones. Two reference mixtures serve as comparisons: a conventional rubber powder composite SBS (styrene–butadiene–styrene triblock)-modified asphalt mixture (CR-SBS) and an SBS-modified asphalt mixture (SBS). A comparative experiment was conducted between the two materials and the SBS-modified asphalt mixture (ACR-SBS) compounded with desulfurized rubber powder. High-temperature stability was tested by the rutting test, low-temperature crack resistance by the beam bending test, and water stability by the immersion Marshall and freeze–thaw splitting tests. Life cycle carbon emissions and economic costs were quantified from raw material acquisition to construction. The results show that desulfurized rubber powder composite with ACR-SBS delivers the most superior overall road performance. However, it also generates the highest life cycle carbon footprint. Its total carbon emission reaches 162,800 kgCO_2_eq, which is 13.7% (19,600 kgCO_2_eq) higher than SBS (143,200 kgCO_2_eq) and 7.7% (11,600 kgCO_2_eq) higher than CR-SBS (151,200 kgCO_2_eq). The total cost of ACR-SBS is 391,000 CNY, which is 1.5% (6000 CNY) higher than SBS (385,000 CNY) and 1.3% (5000 CNY) lower than CR-SBS (396,000 CNY). These findings provide a basis for the selection of high-performance, low-carbon, and economical composite-modified asphalt in severe cold regions.

## 1. Introduction

Asphalt pavement is a primary structural form for urban roads and high-grade highways in China, and its service life directly affects road safety, ride comfort, and maintenance costs [1,2]. During service, the pavement is subjected to multiple stressors, including growing traffic loads, frequent overloading, and extreme climatic conditions [3]. In severe cold regions, pavement distress occurs significantly more often than in other climatic zones [4]. Therefore, the pavement material must have good road performance [5]. Additionally, transportation difficulties in some severe cold regions compromise the delivery and storage stability of modified asphalt, introducing notable logistical constraints and thermal segregation risks [6], leading to quality fluctuations. Therefore, given these unique climatic and environmental conditions, developing high-performance composite-modified asphalt with wide temperature adaptability has become an urgent technical challenge for high-grade highway asphalt pavement construction in these regions.

To mitigate pavement distresses in severe cold climates, polymers such as SBS and crumb rubber are routinely incorporated into asphalt binders [7]. Although SBS profoundly enhances thermal susceptibility and elastic recovery, its stringent manufacturing demands and high costs hinder ubiquitous application [8]. Conversely, rubberized asphalt offers excellent resistance to low-temperature cracking and moisture damage; however, its excessive viscosity and tendency to segregate severely compromise high-temperature stability and workability [9,10]. Consequently, single-modifier systems often fail to concurrently satisfy the rigorous demands of high-grade highways in extreme environments [11]. This limitation has driven contemporary research toward the development of cost-effective, eco-friendly composite modifications [12,13]. While incorporating standard rubber yields considerable economic and recycling benefits, the resulting pavement performance frequently falls short of premium standards [14,15]. Therefore, modifying base asphalt with a combination of SBS and activated rubber powder not only elevates the comprehensive service performance but also effectively mitigates the phase segregation typical of conventional rubber asphalt, presenting substantial engineering potential [15,16,17].

Concurrently, environmental regulations regarding greenhouse gas emissions within the paving sector have become progressively more stringent. Life cycle assessment (LCA) techniques are frequently adopted to measure the carbon footprint of paving materials, encompassing phases from material extraction and plant production through to field paving [18,19]. Notably, the activation treatment of crumb rubber generally introduces extra processing stages, potentially resulting in elevated carbon emissions for activated rubber-modified binders compared to their conventional rubber-modified counterparts [20,21]. Furthermore, recent specialized investigations have begun to construct tailored LCA models and cost–benefit frameworks specifically focusing on desulfurized rubber pavement mixtures to balance their manufacturing environmental premiums against long-term benefits [22,23]. Relying solely on mechanical performance evaluations to judge a material can be somewhat limited. Thus, integrating a full-life-cycle carbon inventory is generally recommended to obtain a more balanced understanding of a mixture’s true engineering viability. In parallel, economic expenditure remains a fundamental factor in pavement engineering decisions and merits careful integration into the overall evaluation. While rubber-composite-modified asphalts typically present initial raw material savings relative to SBSMA [24], a holistic economic assessment must also account for the auxiliary expenses tied to rubber activation, fluctuations in plant mixing costs driven by altered energy demands, and potential increases in paving expenses due to greater compaction resistance. Ultimately, conducting both detailed environmental and economic analyses is vital for a robust assessment of the practical applicability of composite-modified asphalt mixtures.

Existing research primarily focuses on either isolated macro-mechanical performance improvements or the single-dimensional environmental indicator tracking of desulfurized rubber. There is a distinct research gap regarding how the coupled mechanism of polymer–rubber interaction simultaneously influences the engineering, environmental, and economic nexus. Therefore, This study takes ACR-SBS as the primary research object, with CR-SBS and SBS serving as controls. On this basis, the high-temperature stability, low-temperature crack resistance and water stability of the three asphalt mixtures were systematically analyzed. At the same time, the carbon emission levels of the three mixtures in the whole life cycle are quantified to evaluate their low-carbon potential. Finally, considering the practical engineering application of materials, the economic costs of the three mixtures are analyzed. By comprehensively comparing the road performance, low-carbon potential and economic cost of the three asphalt mixtures, this study aims to provide scientific basis and reference suggestions for the rational selection and engineering promotion of composite-modified asphalt in severe cold regions.

## 2. Methodology

**Figure 1:**
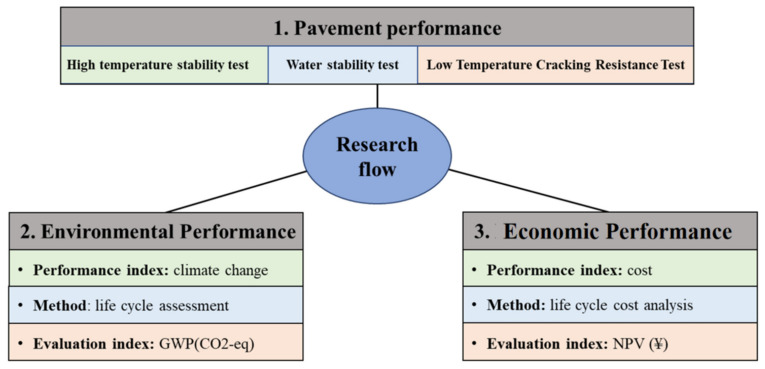
Research flow chart.

To comprehensively evaluate the engineering viability of ACR-SBS, this study conducted a systematic comparative analysis against CR-SBS and SBS across three critical dimensions: technical pavement performance, environmental life cycle impact, and economic feasibility. These three elements are not isolated from each other. Road surface performance analysis serves as the foundation, providing real-time technical condition data of the pavement; without accurate performance predictions, it is impossible to precisely estimate future maintenance requirements and costs. Life cycle assessment applies to bridges, quantifying periodic costs throughout their life cycle based on performance analysis results. Economic evaluation provides the objective basis for decision-making by integrating factors such as time value to achieve optimal life cycle cost minimization. The overall research framework is illustrated in Figure 1.

### 2.1. Materials and Sample Preparation

#### 2.1.1. Materials

(1) Base asphalt

The pristine binder selected for this research was Zhenhai 90# asphalt, which was provided by Gansu Road & Bridge Construction Group Co., Ltd., Lanzhou, China. Its baseline physical characteristics were fully tested by the authors in the laboratory in accordance with the Standard Test Methods of Bitumen and Bituminous Mixtures for Highway Engineering (JTG E20-2011 [25]), with its baseline physical characteristics comprehensively evaluated in Table 1.

(2) SBS modifier

For polymer modification, Linear 1301 SBS produced by Yueyang Petrochemical Co., Ltd. (Yueyang, Hunan, China). was utilized at a fixed dosage of 3% by weight. Both the base asphalt and the SBS modifier were sourced directly from the Gansu Provincial Highway Planning, Survey, and Design Institute, with the fundamental mechanical specifications of the SBS summarized in Table 2. The basic performance indicators of the SBS modifier listed in Table 2 were provided by the Gansu Provincial Highway Planning, Survey, and Design Institute.

(3) Rubber powder

Table 3 outlines the compositional attributes of the crumb rubber incorporated into the matrix, which was provided by Gansu Road & Bridge Construction Group Co., Ltd., Lanzhou, China. These baseline physical and chemical attributes were specified in the technical data sheets provided by the material supplier and cross-checked by the authors following standardized verification protocols. A standardized dosage of 15% was maintained across all rubberized mixtures, processed in a twin-screw extruder at 220 °C, and subjected to oil extraction at 10%, with the desulfurized variant exhibiting a measured solubility of 30%.

(4) Aggregate

Furthermore, the crushed mineral aggregates, categorized into coarse and fine fractions, were procured from Zhongtian Construction Group Co., Ltd., Hangzhou, China. Their morphological and mechanical integrity was rigorously verified against standard thresholds, as detailed in Table 4 and Table 5. The size intervals (2.36–4.75 mm, 4.75–9.50 mm, and 9.5016.0 mm) denote the specific mineral particle size fractions of the raw aggregate stockpiles prior to blending.

#### 2.1.2. Preparation of Composite-Modified Asphalt Mixture

**Figure 2:**
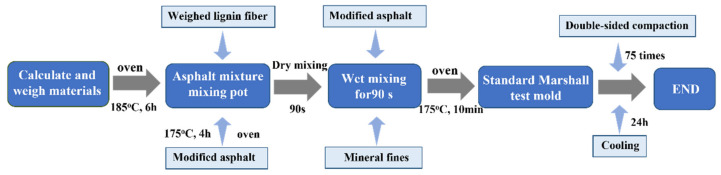
The preparation process of asphalt mixture.

Following the volumetric design protocols outlined in the JTG F40 specifications, the optimal asphalt–aggregate ratios were established at 6.0%, 6.2%, and 6.6% for the SBS, ACR-SBS, and CR-SBS groups, respectively, targeting an air void content of 4.0% alongside standardized stability and flow benchmarks. The sequential compounding, temperature control, and mixing procedures adopted to ensure the uniform dispersion of these modifiers within the asphalt matrix are visually depicted in Figure 2. The mixing process of the asphalt mixture is illustrated as follows. First, calculate and weigh the raw materials, and then preheat them in an 185 °C oven for 6 h. Next, add the weighed desulfurized rubber powder to the SBS-modified asphalt and place the mixture into a mixing bowl; blend at 175 °C for 90 s as a dry mix, followed by adding mineral powder and composite-modified asphalt for wet mixing. Finally, maintain the mixture at 175 °C for 10 min before pouring it into standard Marshall test molds for double-sided compaction and cooling.

### 2.2. Methods on Pavement Performance

The comprehensive pavement performance of ACR-SBS—specifically its resilience to high-temperature deformation, low-temperature cracking, and moisture-induced damage—was evaluated through a suite of standard laboratory protocols, including wheel tracking, immersion Marshall, freeze–thaw splitting, and beam bending tests.

(1) High-temperature stability test

To assess the capacity of the asphalt mixture to withstand cumulative vehicular loading without undergoing significant permanent deformation, wheel tracking tests were conducted to explore the rutting resistance of ACR-SBS. The test temperature was set at 60 °C, with a wheel pressure of 0.7 ± 0.5 MPa. Slabs measuring 300 mm × 300 mm × 50 mm were fabricated using a dedicated roller compactor, sized appropriately for the aggregate gradation. To ensure statistical reliability and eliminate accidental experimental errors, three parallel square slab specimens were prepared and tested for each mixture category, with the dynamic stability (DS) calculated as the mean value. The high-temperature structural integrity was quantified using dynamic stability (DS), defined as the number of wheel passes required to induce 1 mm of rut depth during the final 15 min of the loading cycle.

(2) Water stability test

Moisture susceptibility evaluations are crucial for understanding a mixture’s resistance to binder stripping, raveling, and structural degradation caused by water infiltration. To ascertain the durability of ACR-SBS against such hydrothermal distresses, both immersion Marshall and freeze–thaw splitting tests were executed to simulate field erosion and seasonal freeze–thaw weathering. The Marshall experiment specimen dimensions were 101.6 mm in diameter and 63.5 mm in height; the test was conducted with a holding time of 48 h and a loading rate of 50 mm/min. The mixture’s resistance to moisture damage was objectively indexed through the retained Marshall stability (MS_0_) and the tensile strength ratio (TSR). For each distinct asphalt mixture formulation, a batch of four identical cylindrical specimens was fabricated using a standard Marshall compactor (75 blows per side). The reported parameters represent the mathematical average of these four independent replicates to guarantee data reproducibility.

(3) Low-Temperature Cracking Resistance Test

To investigate the susceptibility of ACR-SBS to thermal shrinkage cracking in severe cold environments, low-temperature beam bending tests were employed. Initially, compacted slabs were sawn into prismatic beam specimens with dimensions of 250 mm × 30 mm × 35 mm. Prior to mechanical loading, these beams were conditioned in an environmental chamber at −10 °C for 60 min. The test loading rate was 50 mm/min. Subsequently, a CMT5105 universal testing machine was utilized to apply the load. The low-temperature cracking resilience of ACR-SBS was comprehensively characterized by deriving the flexural tensile strength, maximum flexural strain, and flexural stiffness modulus from the failure data.

### 2.3. Life Cycle Assessment Method

Life cycle assessment is a method of environmental impact assessment based on international standard ISO 14040 [26]. Generally, this life cycle assessment involves four steps: goal and scope definition, inventory analysis, impact assessment, and interpretation of results [27]. To mathematically capture the carbon footprint, the greenhouse gas calculation models were adopted from referenced studies [28,29], the specific calculation formulas are shown in Equations (1) and (2).

(1) $$E_{R}=\sum (M_{i}\times P_{i})$$

where *E_R_* is the CO_2_ emissions corresponding to raw materials, *kgCO*_2_*eq*; *M_i_* is the mass of work corresponding to raw materials from category *i*, *t*; and *P_i_* is the CO_2_ emission factor corresponding to raw material type *i*, *kgCO*_2_*eq/t*.

(2) $$E_{P}=\sum \sum m_{j}Q_{j}P_{j}GWP_{k}$$

where *E_P_* is the total carbon emissions of mixture production, *m_j_* is energy *j* consumption, *Q_j_* is the unit heat quantity of energy *j*, *P_j_* is the carbon emission factors of energy *j*, and *GWP_k_* is the *GWP* of the greenhouse gas *k*.

(1) Goal and scope definition

**Figure 3:**
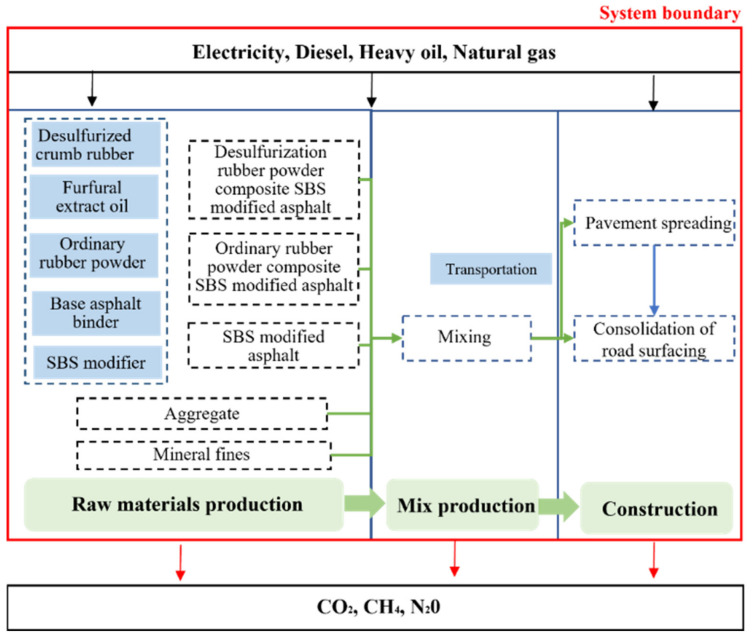
System boundary.

This study analyses the global warming impacts of the production phase of ACR-SBS materials based on a cradle-to-gate perspective. Meanwhile, in order to highlight the environmental impact of ACR-SBS, CR-SBS and SBS are included for comparison. The computational parameters and baseline information have been systematically reorganized in close alignment with established reporting frameworks for construction materials. The comprehensive data streams are explicitly categorized into three sequential phases to ensure maximum transparency: (i) raw material production, (ii) mix production, and (iii) construction. For each individual phase, the corresponding input consumption values, reference standards, and specific carbon emission intensity factors are clearly mapped and explicitly cited. The system boundary of this study was defined as the wearing course of a first-class highway test section, with dimensions of 1000 m in length, 7.5 m in width, and 0.04 m in thickness. The system boundary is shown in Figure 3.

Given that CO_2_, CH_4_, and N_2_O are established as the primary anthropogenic drivers of global warming [30,31,32], this study explicitly quantified their respective emissions during the material production stages for all three mixtures. These outputs were subsequently converted into carbon dioxide equivalents to facilitate standardized comparisons. Furthermore, reflecting the prolonged atmospheric residence time of CO_2_, a conventional 100-year time horizon was adopted for the global warming potential analysis [33,34]. The cumulative carbon emissions encompassing all evaluated life cycle stages are determined utilizing Equation (3).

(3) $$E=E_{R}+E_{M}+E_{C}$$

where *E* is the total greenhouse gas emissions, *kgCO*_2_*eq*; *E_R_* is the greenhouse gas emissions during the raw material production stage, *kgCO*_2_*eq*; *E_M_* is the greenhouse gas emissions during the mix production stage, *kgCO*_2_*eq*; and *E_C_* is the greenhouse gas emissions during the construction stage, *kgCO*_2_*eq*.

(2) Life cycle inventory

The life cycle inventory phase essentially comprises gathering primary and secondary information to evaluate the physical inputs and outputs associated with processes inside the system boundary [35]. Within this investigation, data corresponding to the cradle-to-gate scope were collated from standard specifications, previous studies, and machinery assessments. The specific materials utilized in this analysis are cataloged in Table 6 [29,36,37,38,39,40,41].

Considering the similarity of the preparation process of composite-modified asphalt and modified asphalt, this study only considers the difference in materials in the calculation of carbon emissions of composite-modified asphalt and modified asphalt, and does not consider the difference in energy consumption. At the same time, considering that there is still a distance from material production to mixture mixing, this study is based on the engineering construction projects in Gansu Province, China, and the data of materials are obtained as shown in Table 7, Table 8 and Table 9 [29].

### 2.4. Life Cycle Cost Analysis Method

Parallel to greenhouse gas evaluations, financial feasibility remains a pivotal consideration in the design and construction of asphalt pavements. The scope of the financial appraisal in this research primarily incorporates expenses tied to raw material procurement, logistics and transit, energy utilization, and the deployment of paving machinery. Empirical unit pricing for these components was sourced directly from a regional infrastructure project situated in Gansu Province, China. To systematically quantify the total financial expenditure, the overarching cost model is mathematically formulated as Equation (4).

(4) $$C=C_{R}+C_{M}+C_{C}$$

where *C* is the total cost, in CNY; C*_R_* is the cost of the raw material production stage, in CNY; C*_M_* is the cost of the mixture production stage, in CNY; and *C_C_* is the cost of the construction production stage, in CNY.

## 3. Results and Discussion

### 3.1. Analysis of Pavement Performance of Composite-Modified Asphalt Mixture

(1) High-temperature stability

**Figure 4:**
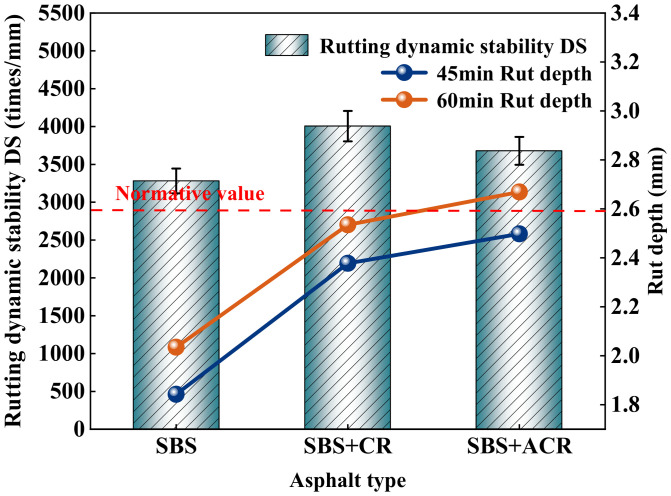
Rutting test results.

In this study, the rutting test was carried out on three kinds of modified asphalt mixtures: ACR-SBS, CR-SBS and SBS. The test results are shown in Figure 4.

Figure 4 shows the rutting test results of the three modified asphalts. It can be seen from Figure 4 that the dynamic stability indexes of the three asphalt mixtures can meet the requirements of Chinese specifications. While CR-SBS exhibits the highest high-temperature dynamic stability owing to its intact cross-linked rubber networks, this single-dimensional advantage often compromises low-temperature flexibility. The chemical desulfurization in ACR-SBS slightly degrades the high-temperature shearing resistance, yet it yields a more balanced and optimized comprehensive road performance. This engineering trade-off mitigates the common brittle cleavage failures of traditional rubberized asphalt, establishing a highly resilient material balanced across conflicting high-temperature, low-temperature, and moisture performance criteria. Comparing the three different asphalt mixtures shows that the incorporation of rubber powder improves the dynamic stability of asphalt mixture. The dynamic stability of ACR-SBS and CR-SBS is 12.1% and 22.1% higher than that of SBSMA, respectively. Meanwhile, the desulfurization process of the rubber powder appears to compromise the dynamic stability of the mixture. This observation suggests that activating the crumb rubber prior to its incorporation might diminish the overall rutting resistance of the asphalt.

(2) Low-Temperature Cracking Resistance Test

**Figure 5:**
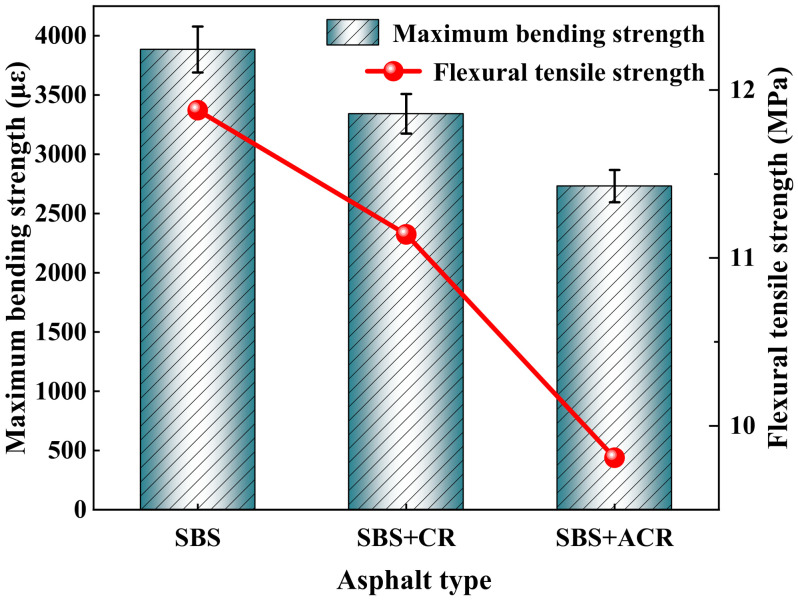
Results of beam bending test.

In this study, the low-temperature trabecular bending test was carried out on three kinds of modified asphalt mixtures: ACR-SBS, CR-SBS and SBS. The experimental results are shown in Figure 5.

Figure 5 shows the experimental results of the trabecular bending test of the three asphalt mixtures. The line chart illustrates the material’s tensile strength, measured in units of tensile strength, while the bar chart represents its maximum bending strength, expressed in microstrain units. It can be seen from Figure 5 that the flexural strength of SBS is the largest, and the flexural strength of ACR-SBS is the smallest. By comparing the three different asphalt mixtures, it can be seen that the addition of rubber powder reduces the flexural tensile strength of asphalt mixture. When comparing the distinct rubber powder variants, mixtures incorporating desulfurized rubber generally exhibit a reduced flexural tensile strength relative to those utilizing conventional rubber powder. This phenomenon can primarily be attributed to the elevated viscosity inherent in standard rubber-modified binders, which typically necessitates a higher asphalt–aggregate ratio. Consequently, this leads to a thicker asphalt film and a greater volume of free asphalt distributed within the mixture matrix. In cold environments, the augmented cohesive forces supplied by this excess free binder tend to enhance the overall flexural tensile resistance of the composite.

(3) Water stability test

**Figure 6:**
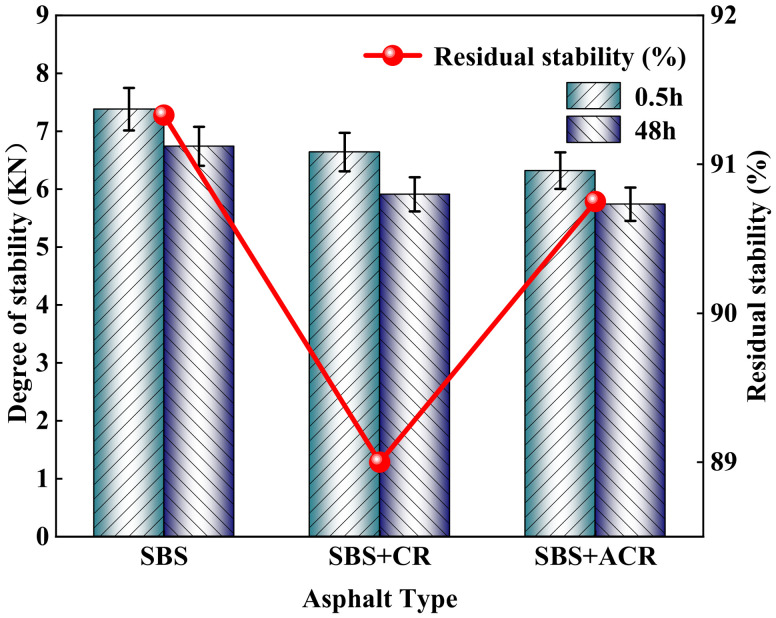
Results of immersion Marshall test.

In this study, the three modified asphalt mixtures of ACR-SBS, CR-SBS and SBS were subjected to the immersion Marshall test. The test results are shown in Figure 6.

Figure 6 shows the immersion Marshall test results for the three asphalt mixtures. It can be seen from Figure 6 that the residual stability of the three asphalt mixtures is greater than 80%, which meets the requirements of the specification. The maximum stability of the three asphalt mixtures is SBS, and the minimum is ACR-SBS. In general, the stability value serves as a key indicator of an asphalt pavement’s capacity to withstand irreversible deformation and rutting in elevated temperature environments, alongside its load-bearing competence and mechanical response under external stresses. Consequently, this stability metric is inherently correlated with the high-temperature resilience of the asphalt mixture. For SBS, the stability is greater than that of the other two kinds of composite-modified asphalt mixtures. This is because the factors affecting the performance of the asphalt mixture are not only asphalt performance, but also gradation type, asphalt content, porosity, environmental climate and many other factors. Among them, the asphalt–aggregate ratio of the two composite-modified asphalt mixtures is greater than that of the SBS. Generally, the magnitude of the asphalt–aggregate ratio is a critical factor governing the skeletal framework and the aggregate interlock within the mixture. For the two composite-modified mixtures, this ratio is comparatively elevated, contributing to an increased presence of free asphalt distributed among the mineral particles. Such an accumulation tends to compromise the integrity of the aggregate skeleton, subsequently diminishing the internal interlocking friction and thereby limiting the mixture’s high-temperature stability.

It can be seen from Figure 6 that the maximum residual stability of the three asphalt mixtures is SBS, and the minimum is CR-SBS. Regarding the influence of rubber powder variations, mixtures incorporating desulfurized rubber generally exhibit an elevated residual stability compared to those containing conventional crumb rubber. This finding implies that the rapid degradation of the residual stability for the CR-SBS mixture is governed by the hydrophobic and chemically inert surface of unactivated rubber particles, which forms weak physical interfaces with the binder. When exposed to hydrothermal erosion, water molecules easily penetrate these interface boundary zones, leading to severe binder stripping. Conversely, the degradation processes associated with desulfurization break the vulcanization networks, producing small-molecular fractions and highly active radical groups. This chemically promotes superior interfacial adhesion and chemical bonding between the asphalt matrix and the mineral aggregates, effectively mitigating moisture-induced degradation at the binder–aggregate boundary and preventing the rapid decline seen in the CR-SBS group.

**Figure 7:**
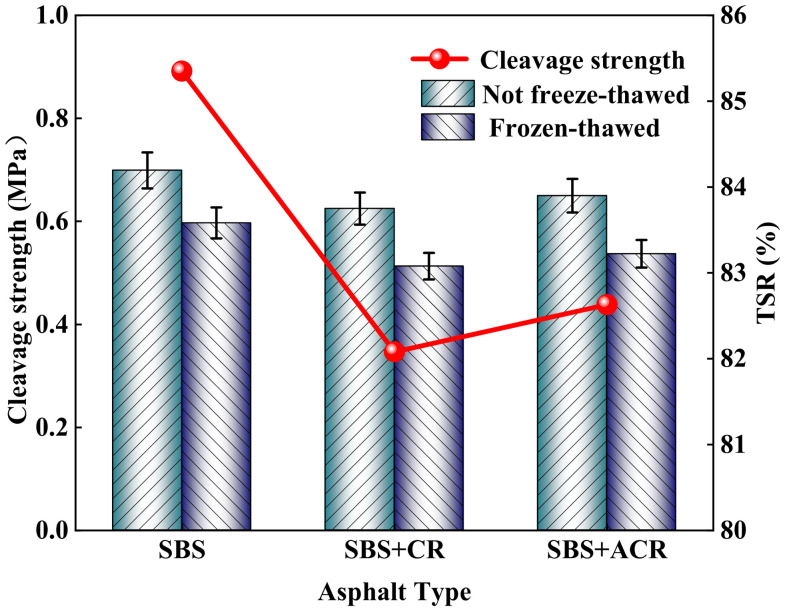
Results of freeze–thaw splitting test.

In this study, freeze–thaw splitting tests were carried out on three kinds of modified asphalt mixtures: ACR-SBS, CR-SBS and SBS. The test results are shown in Figure 7.

Figure 7 shows the results of freeze–thaw splitting tests of the three modified asphalt mixtures. It can be seen from Figure 7 that the TSR values of the three asphalt mixtures are all greater than 80%. This shows that the three modified asphalt mixtures still maintain good water stability under freeze–thaw conditions. The splitting strength of the three modified asphalt mixtures without freeze–thaw and after freeze–thaw is the largest for SBS, and the smallest is the CR-SBS. This behavior can primarily be attributed to the fact that, at ambient temperatures, a higher asphalt–aggregate ratio generally results in a thickened asphalt film. Consequently, the relative proportion of structural asphalt engaging with the aggregates tends to diminish. Such a reduction subsequently impairs the cohesive properties of the mixture, ultimately leading to a decline in its splitting flexural strength. The largest freeze–thaw splitting strength ratio of the three asphalt mixtures is SBS, and the smallest is CR-SBS. These results suggest that while the direct inclusion of crumb rubber tends to negatively impact the moisture resistance of the mixture, applying a desulfurization treatment to the rubber powder generally serves to enhance its overall water stability.

To establish a comprehensive understanding of the macro-performance evolutions, the observed mechanical responses of the SBS, CR-SBS, and ACR-SBS mixtures are critically contextualized with recent literature benchmarks regarding rubber–polymer composite asphalt systems. The experimental matrix reveals a distinct performance trade-off dominated by the rubber chemical desulfurization intensity. Specifically, compared with the raw crumb rubber-modified mixture (CR-SBS), the desulfurized variant (ACR-SBS) exhibits a marginal reduction in high-temperature rutting resistance (dynamic stability). This variation aligns with the findings of previous researchers [43], who noted that the severing of cross-linked vulcanization networks restores rubber molecular mobility but slightly compromises elastic recovery under high thermal shearing. Crucially, however, this subtle high-temperature sacrifice is compensated for by a profound leap in low-temperature cracking strain and moisture resilience. This mechanism is derived from the superior multi-phase compatibility between the depolymerized rubber chains and the SBS/bitumen matrix [38], which alleviates the microstructural stress concentration typical of traditional un-plasticized rubberized asphalt, shifting the structural failure mode from brittle cleavage to ductile yielding.

### 3.2. Analysis of Carbon Emission of Composite-Modified Asphalt Mixture

Based on the project in Northwest China, this study selected a 0.04-meter-thick pavement wear layer. The carbon emissions of ACR-SBS, CR-SBS and SBS were compared and analyzed using the life cycle assessment method. The quantitative analysis of carbon emissions involves the raw material production stage, mixture production stage and construction stage.

(1) Raw material acquisition stage

**Figure 8:**
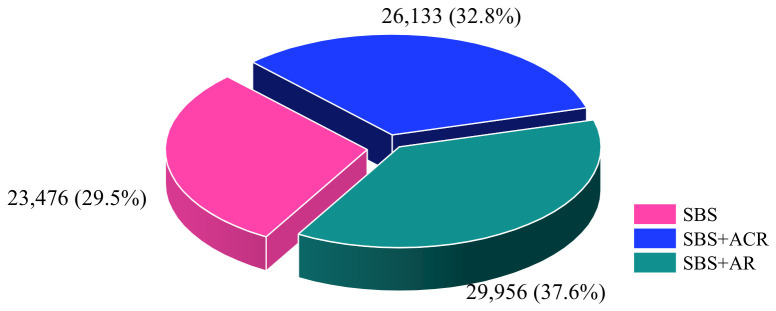
Carbon emissions of raw material acquisition stage.

The carbon emissions in the raw material acquisition stage of SBS, ACR-SBS and CR-SBS were analyzed, as shown in Figure 8.

Figure 8 shows the proportion of carbon emissions of the three asphalt mixtures in the raw material acquisition stage. It can be seen from Figure 8 that ACR-SBS has the largest proportion of carbon emissions. From the perspective of greenhouse gases, the environmental benefits of SBS are better than the carbon emissions of the other two asphalt mixtures.

**Figure 9:**
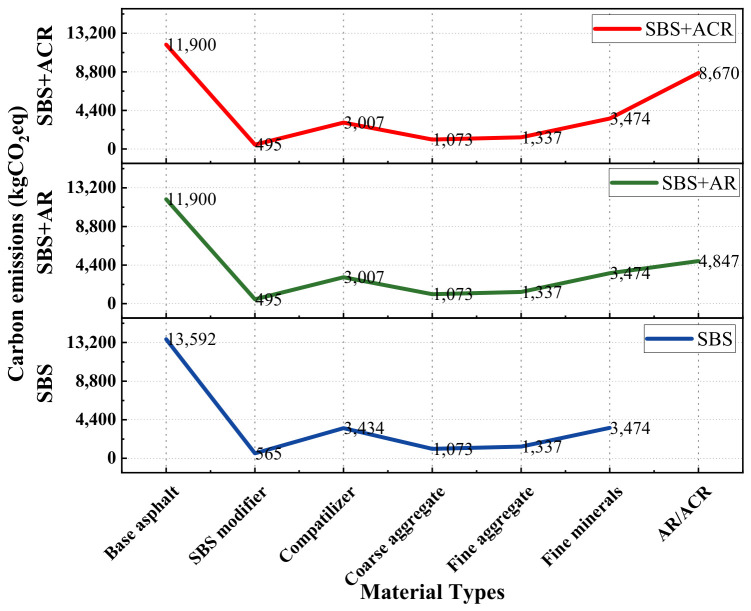
Details of carbon emissions in the raw material acquisition stage.

The details of carbon emissions in the raw material acquisition phase are shown in Figure 9. It can be seen from Figure 9 that the difference between the three asphalt mixtures is the carbon emission of rubber powder. Generally, incorporating rubber powder tends to elevate the overall carbon footprint of the asphalt mixture. Concurrently, as illustrated in Figure 9, the desulfurization process applied to the rubber powder appears to further contribute to these emissions. This indicates that the greenhouse gases released during the raw material acquisition phase are not driven exclusively by the volume of materials consumed, but also by the processing complexity involved in obtaining them.

(2) Mixture production stage

**Figure 10:**
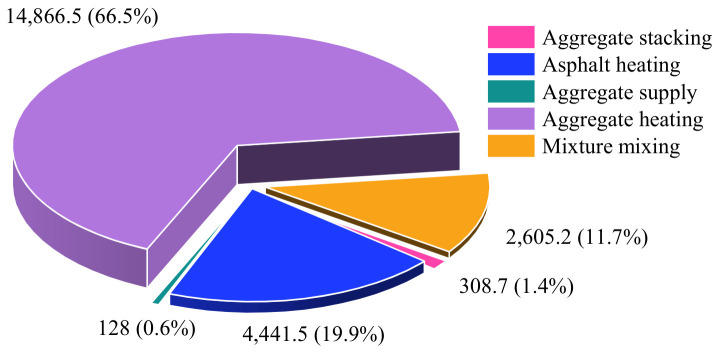
Carbon emissions of each asphalt mixture in the production stage of the mixture.

The production stage of the mixture involves five processes: aggregate storage, aggregate supply, aggregate heating, asphalt heating and mixing. The study quantified the greenhouse gases produced by the five processes, as shown in Figure 10.

As illustrated in Figure 10, aggregate heating represents the most significant contributor to greenhouse gas emissions, being responsible for approximately 66.5% of the total carbon footprint during the mixture production phase. Conversely, the aggregate storage step accounts for the lowest share, at roughly 1.4%. It is important to note that these emission values and proportional distributions are exactly identical across all three investigated mixtures (SBS, CR-SBS, and ACR-SBS). This parity stems from the practical highway engineering reality that all three mixtures are processed using the same batch-mix plant equipment under standardized manufacturing parameters, where the fuel oil consumption for heating aggregates and electricity inputs for mechanical mixing remain uniform per ton of material. The target mixing temperature thresholds do not vary drastically enough to trigger discrete inventory quotas in this phase; hence, the specialized environmental penalties induced by rubber desulfurization and polymer modifications are entirely isolated within the raw material acquisition stage. The primary driver of these emissions throughout the production stage is inherently tied to energy consumption, which typically relies on diesel, heavy oil, and electricity. The disproportionately high emissions observed during the aggregate heating process can generally be attributed to both the substantial volume of fuel required and the relatively high carbon emission factors associated with these specific energy sources.

(3) Construction stage

**Figure 11:**
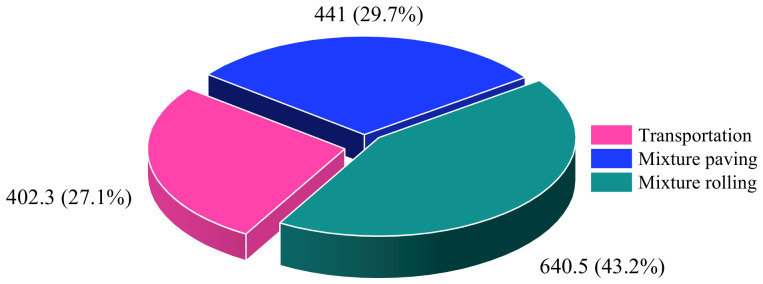
Carbon emissions of each asphalt mixture in the construction stage.

In this study, there are three processes included in the construction stage, which are mixture transportation, mixture paving and mixture compaction. The greenhouse gases of these three processes were calculated, and the results are shown in Figure 11.

According to the data presented in Figure 11, mixture compaction accounts for a substantial proportion of greenhouse gas emissions during the construction phase, representing approximately 43.2% of the stage’s total footprint. In contrast, hauling operations contribute a relatively smaller share at 27.1%. Notably, transportation-related emissions are subject to considerable variability. Assuming a standardized mixture mass, this carbon output is predominantly dictated by the hauling distance. Because the transit routes in the current study were relatively short, the corresponding energy expenditure remained minimal, thereby keeping the overall construction-stage emissions comparatively low.

**Figure 12:**
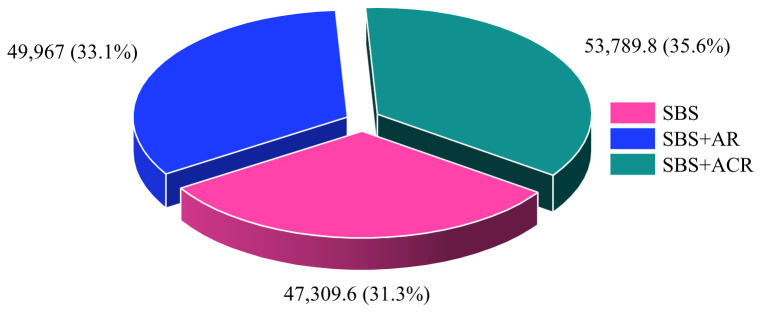
Comparison of total carbon emissions of modified asphalt mixture.

In summary, the total carbon emissions of SBS, CR-SBS and ACR-SBS in raw material acquisition, mixture production and construction stage were quantitatively calculated, as shown in Figure 12.

It can be seen from Figure 12 that the ACR-SBS has the largest total greenhouse gas generated in the three stages of raw material acquisition, mixture production and construction. Among the three asphalt mixtures, SBS produces the smallest amount of greenhouse gas emissions. Comparing the greenhouse gas emissions of the three asphalt mixtures, it can be seen that compared with the SBS, the other two asphalts are mainly mixed with rubber powder. In terms of environmental benefits, SBS has more superior environmental benefits than the other two asphalt mixtures. The ACR-SBS has poor environmental benefits. The greenhouse gas emission of ACR-SBS is 7.7% more than that of CR-SBS. The greenhouse gas produced by ACR-SBS is 13.7% more than that produced by SBS. For the two rubber powder composite-modified asphalt mixtures (ACR-SBS and CR-SBS), the process of desulfurization rubber powder treatment is more complicated than that of ordinary rubber powder. Therefore, the greenhouse gas produced by ACR-SBS is more than that produced by CR-SBS.

The established LCA framework operates within a cradle-to-gate boundary (terminating at the pavement construction gate), which inherently omits the environmental impacts during the long-term operation, routine maintenance, and ultimate reclamation phases. This boundary limitation is selected because the precise multi-year material degradation, environmental weathering, and structural cracking propagation under real-world traffic cannot be reliably captured through accelerated laboratory aging methods, making further life cycle extrapolation highly speculative. To overcome this constraint and deepen the environmental tracking, our research team is currently launching a 500 m high-grade highway field test section. The subsequent long-term in situ monitoring—including surface distress auditing and structural capacity tracking via falling weight deflectometers (FWD)—will systematically harvest real-world pavement durability datasets. This empirical feedback will provide the baseline required to extend the current greenhouse gas inventory into a comprehensive, full-service timeline cradle-to-grave life cycle model.

### 3.3. Analysis of Economic Cost of Composite-Modified Asphalt Mixture

**Figure 13:**
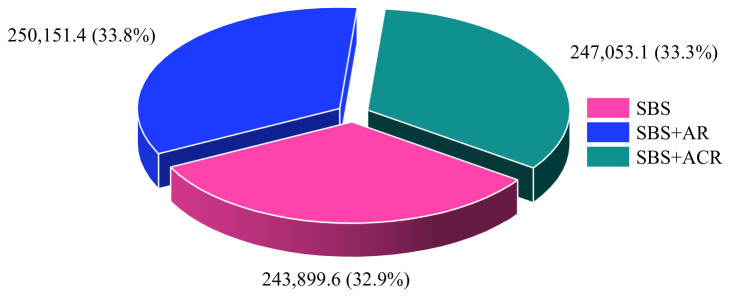
Comparison of economic cost of modified asphalt mixture.

In this study, the economic costs of SBS, CR-SBS and ACR-SBS were analyzed. The analysis stage includes the raw material acquisition, mixture production and construction stages, as shown in Figure 13.

It can be seen from Figure 13 that the economic cost of CR-SBS is the largest, while the economic cost of SBS is the smallest. However, by comparing the economic costs of the three asphalt mixtures, it can be seen that the economic cost of CR-SBS is 1.3% more than that of ACR-SBS. The economic cost of CR-SBS is 2.6% higher than that of SBS.

**Figure 14:**
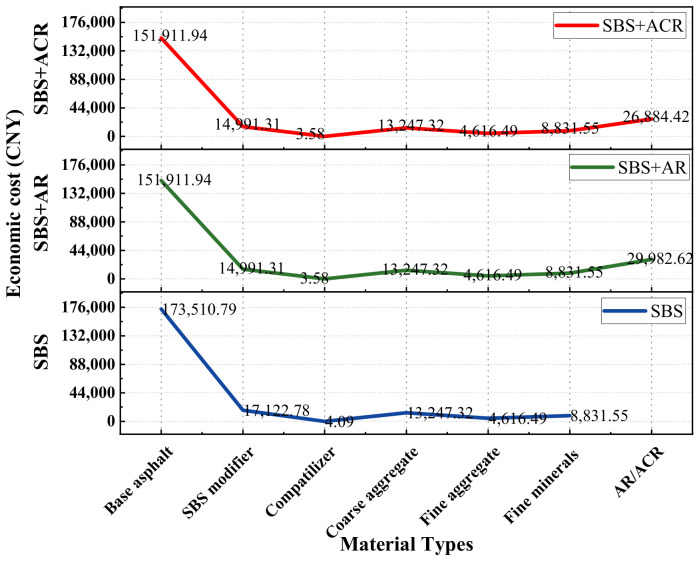
Comparison of economic cost details of modified asphalt mixture.

The details of the economic cost of raw materials were calculated, and the results are shown in Figure 14.

It can be seen from Figure 14 that due to the incorporation of rubber powder, the economic cost of the rubber powder composite-modified asphalt mixtures is higher than that of SBS. The economic cost of ACR-SBS is 1.5% higher than that of SBS. The economic cost of CR-SBS is 2.9% higher than that of SBS. A comprehensive comparison of the economic costs of the three asphalt mixtures shows that the economic benefits of SBS are better, while the economic costs of CR-SBS are worse than the other two.

It is vital to clarify that the comparable economic total cost among the three mixtures is not an artifact of rough data estimation, but a direct consequence of the volumetric design optimization. Although the chemical desulfurization process introduces extra processing costs, equipment depreciation, and compatibility additives, the optimal asphalt–aggregate ratio of ACR-SBS (6.2%) is lower than that of CR-SBS (6.6%). In highway infrastructure projects, the massive consumption of premium bitumen binders represents the primary financial weight. Therefore, the significant reduction of 0.4% in the overall binder dosage successfully cushions and offsets the manufacturing cost premiums of rubber activation, leading to a highly competitive market price compared to both reference groups. This observed carbon emission trajectory is highly consistent with recent prospective life cycle frameworks for advanced crumb rubber pretreatment technologies in asphalt pavements [38].

To provide a clear, direct, and unified baseline for cross-dimensional comparison across the technical, environmental, and economic indexes, the quantitative laboratory results and full-stage evaluation indicators of the three investigated asphalt mixtures (SBS, CR-SBS, and ACR-SBS) are systematically consolidated in Table 10. This centralized dataset presents the calculated experimental mean values paired with their respective standard deviations, establishing a reliable multi-criteria dataset to further dissect the subtle trade-offs and balancing mechanisms within the pavement performance–environmental–cost nexus.

## 4. Conclusions

In this study, ACR-SBS was taken as the research object, and CR-SBS and SBS were taken as the control. The high-temperature stability, low-temperature crack resistance and water stability of the three mixtures were analyzed. At the same time, the life cycle assessment method was used to quantify the carbon emission level of the three mixtures in the whole process of raw material acquisition, mixing and construction. Combined with the economic cost analysis, the road performance, low carbon potential and economic cost of the three asphalt mixtures were comprehensively compared. To bridge the gap between laboratory findings and engineering practice, the research team is actively planning and coordinating the construction of a 500-meter field pavement test section on a representative high-grade highway corridor. Future investigations will systematically execute long-term in situ tracking tests—encompassing surface distress field surveys, structural capacity evaluations via falling weight deflectometers (FWD), and annual field core extractions—to monitor the real-world microstructural degradation and macro-pavement durability of the desulfurized rubber powder composite SBS-modified asphalt mixture over an extended service timeline. The main conclusions of this paper are as follows:

(1) ACR-SBS delivers a balanced and optimized comprehensive pavement performance. Its high-temperature stability is 12.1% higher than that of SBS, despite a slight reduction compared to CR-SBS due to rubber activation. In terms of low-temperature crack resistance and moisture stability, ACR-SBS ranks second only to SBS and outperforms CR-SBS, successfully mitigating the brittleness of traditional rubberized asphalt.
(2) ACR-SBS has the highest carbon emissions in the whole life cycle. The total greenhouse gas emissions of ACR-SBS in the three stages of raw material acquisition, mixture production and construction are 13.7% higher than that of SBS, and 7.7% higher than that of CR-SBS.
(3) The economic cost of ACR-SBS is between those of SBS and CR-SBS. The economic cost of ACR-SBS is 1.5% higher than that of SBS, but 1.3% lower than that of CR-SBS.
(4) The system boundary for carbon footprint accounting in the current research is primarily restricted to the material extraction, plant production, and on-site paving phases. Consequently, downstream processes—such as operational maintenance, periodic repairs, and end-of-life recycling—are not encompassed within this scope. Future investigations could potentially expand these boundaries to encompass a complete cradle-to-grave framework, thereby providing a more holistic assessment of the environmental burdens.

## Figures and Tables

**Table 1:** Basic physical properties of the pristine Zhenhai 90# binder.

Evaluated Parameters		Measured Values
Penetration (25 °C, 100 g, 5 s) (0.1 mm)		87.2
Softening point (°C)		47.4
Ductility (10 °C) (cm)		>100
RTFOF (163 °C, 85 min)	Mass loss (%)	0.12
	Penetration ratio (25 °C) (%)	68
	Ductility (10 °C) (cm)	8.0

**Table 2:** Fundamental properties and structural parameters of the Linear 1301 SBS.

Evaluated Parameters	Measured Values
Tensile strength (MPa)	15.00
300% constant tensile strength (MPa)	2.0
Hardness (Degree A)	≥68.00
Permanent deformation at tear (%)	≤40.00
S/B insertion ratio	3/7

**Table 3:** Compositional and physical attributes of the utilized crumb rubber.

Evaluated Parameters	Measured Values
Relative density (kg/m^3^)	1140
Water content (%)	0.490
Iron content (%)	0.010
Fiber content (%)	0.100
Ash content (%)	7.190
Acetone extractives (%)	6.310
Carbon black content (%)	31.00
Rubber hydrocarbon content (%)	49.00

**Table 4:** Engineering properties of the coarse mineral aggregates.

Evaluated Parameters	Measured Values		
	2.36–4.75	4.75–9.5	9.5–16
Apparent specific gravity	2.65	2.73	2.69
Water absorption (%)	0.70	0.78	0.63
Crushing value (%)	/	/	12.3
Soundness (%)	6	6	5
Los Angeles abrasion value (%)	13.9	12.8	8.4

**Table 5:** Quality assessment metrics of the fine aggregates.

Evaluated Parameters	Measured Values
Apparent specific gravity	2.56
Soundness (>0.3 mm) (%)	19.00
Methylene blue number (g·kg^−1^)	1.60
Sand equivalent (%)	81.00
Angularity (flow time) (s)	38.50

**Table 6:** Material list data.

Name	LCA Data Source
Base asphalt	Feng Ma [36]
Ordinary rubber powder	Haoran Zhu [39]
Desulfurized crumb rubber	Wei Li [38]
SBS modifier	A Riekstins [41]
Compatilizer	Haoran Zhu [42]
Coarse aggregate	Feng Ma [43]
Fine aggregate	Feng Ma [36]
Mineral fines	Feng Ma [36]
Electricity	Feng Ma [36]
Diesel oil	Feng Ma [36]
Heavy oil	Feng Ma [36]

**Table 7:** Investigation the energy consumption of asphalt mixture production.

Position	Aggregate Stacking (Diesel) (L/t)	Asphalt Heating	Aggregate Supply (Diesel) (L/t)	Aggregate Heating	Mixture Mixing (Electricity) (kWh/t)
Gansu Province, China	0.164	35.124 (oil) (kg/t)	0.068	7.054 (oil) (kg/t)	3.95

**Table 8:** Transportation energy metrics for the asphalt mixture.

Position	Transportation (Diesel) (L/km·t)	Load Distance (km)
Gansu Province, China	0.012	16.8

**Table 9:** Construction energy metrics for the asphalt pavement.

Position	Mixture Paving (Diesel) (L/t)	Mixture Rolling (Diesel) (L/t)
Gansu Province, China	0.221	0.321

**Table 10:** Comprehensive experimental results of pavement performance, environment, and cost for the three asphalt mixtures.

Evaluation Indexes	SBS	CR-SBS	ACR-SBS
Dynamic Stability (DS, times/mm)	3281	4005	3679
Flexural Tensile Strength (RB, MPa)	11.88	11.14	9.81
Maximum Flexural Strain (ϵB, μϵ)	3884.12	3341.07	2731.22
Retained Marshall Stability (MS0, %)	91.33	89	90.75
Tensile Strength Ratio (TSR, %)	85.35	82.08	82.63
Total Carbon Emissions (kgCO_2_eq)	47,309.6	49,967.0	53,789.8
Total Financial Cost (CNY)	243,899.6	250,151.4	247,053.1

## Data Availability

The original contributions presented in this study are included in the article. Further inquiries can be directed to the corresponding authors.
